# Development of Electrochemical Oscillation Method for Identification of *Prunus persica, Prunus davidiana*, and *Prunus armeniaca* Nuts

**DOI:** 10.3389/fchem.2020.00748

**Published:** 2020-09-11

**Authors:** Shuai Yan, Yinzi Yue, Lianlin Su, Min Hao, Xiaopeng Wang, Ting Zuo

**Affiliations:** ^1^Suzhou TCM Hospital Affiliated to Nanjing University of Chinese Medicine, Suzhou, China; ^2^School of Pharmacy, Nanjing University of Chinese Medicine, Nanjing, China; ^3^School of Pharmacy, Zhejiang Chinese Medicine University, Hangzhou, China; ^4^School of Pharmacy, Henan University of Chinese Medicine, Zhengzhou, China

**Keywords:** electrochemical oscillation, Belousov–Zhabotinsky, plant identification, herbal medicine, *Prunus* spp.

## Abstract

In this work, an electrochemical oscillation system has been developed using the Belousov–Zhabotinsky reaction. The effect of the combination of each reagent, reaction temperature, and stirring speed on the induction period, oscillating period, and oscillating life were optimized. The nuts of *Prunus persica, Prunus davidiana*, and *Prunus armeniaca* have been widely used for medical purposes. The proposed electrochemical oscillation system was then used for the identification of *P. persica, P. davidiana*, and *P. armeniaca*. Three nuts exhibited very different electrochemical oscillation profiles. The dendrogram was divided into three main principal infrageneric clades. Each cluster only contains one species, suggesting that no outlier was observed in this study. Based on the discussed results, we proposed a simple method for herbal medicine identification.

## Introduction

The fingerprint of traditional herbal medicine refers to the map of common peaks that can mark the characteristics of the traditional herbal medicine after proper pretreatment with a certain analysis method (Xie et al., 2006; Zhu et al., 2016; Wang et al., 2017). It is the visual characterization of the physical and chemical information of the traditional herbal medicine (Donno et al., 2016; Deconinck et al., 2017; Afshar et al., 2020; Fu et al., 2020; Karimi-Maleh et al., 2020a,c; Li et al., 2020; Tavana et al., 2020). The fingerprint of traditional herbal medicine has two characteristics: (1) Through the characteristics of the fingerprint, it can identify the authenticity or origin of the sample. (2) The area or ratio of the main characteristic peaks on the fingerprint can effectively control the quality of traditional herbal medicine.

At present, the common fingerprints of traditional herbal medicine are thin layer chromatography fingerprint (Szeremeta et al., 2017; Sibug-Torres et al., 2019a,b), high-performance liquid chromatography fingerprint (Li et al., 2016; Xue et al., 2017; Esteki et al., 2019), gas chromatography fingerprint (Aliakbarzadeh et al., 2016; Shekari et al., 2018; Huang et al., 2019), high-performance capillary electrophoresis fingerprint (Hou et al., 2019; Sun et al., 2019), high-speed countercurrent chromatography fingerprint (Gan et al., 2016; Wang et al., 2020), ultraviolet fingerprint, infrared fingerprint (Custers et al., 2017; Dai et al., 2019), nuclear magnetic resonance fingerprint (Sun et al., 2018; Flores et al., 2020), mass spectrometry fingerprint (Yang and Deng, 2016; Kharbach et al., 2020), X-ray diffraction fingerprint (Chen et al., 2017; Devi et al., 2017), immunoassay fingerprint (Cui et al., 2019), and DNA fingerprint (Pawar et al., 2017; Selvakumari et al., 2017). These methods can be used for in-depth research on traditional herbal medicine, but proper pretreatment of traditional herbal medicine is required. The pretreatment process will cause the loss of ingredients, so the existing fingerprint is actually only a collection of information on the chemical composition of some herbal medicines, not a complete reflection of the chemical composition of herbal medicines. Therefore, it is a primary task to explore a direct operation method of fingerprints that can be applied to the cluster characterization of traditional herbal medicine chemical components in various phases and dosage forms without pretreatment operations such as separation and purification. The discovery of non-linear chemical phenomena has opened the study of complex systems. Due to the good reproducibility of the oscillating response, its application research is becoming more and more extensive, which provides a new idea and method for solving this problem.

At present, the Belousov–Zhabotinsky (B-Z) oscillation reaction is most widely used in analysis and detection, followed by the copper ion oscillation system (Alfifi et al., 2016; Luiz Fernando Oliveira Maia et al., 2019; Nawabi et al., 2019; Ullah et al., 2019). There are also Bray–Liebhafsky oscillation reaction, Briggs–Rauscher oscillation reaction, peroxidase–oxidase biochemical oscillator (peroxidase–oxidase oscillation system), and liquid membrane oscillator (Mukouyama et al., 2016; Bai et al., 2017; Peng et al., 2017; Chan and Dow, 2019; Ding et al., 2019). The basis for applying chemical oscillation reaction to analysis and detection is that the substance to be tested can interfere with the oscillation reaction. Different chemical oscillation reactions have different characteristics, and the shape of the potential–time (E-t) curve and various characteristic information parameters are also different (Miyazaki et al., 2016; Zhou et al., 2020). Herbal medicines of different types or origins and sources contain different chemical components and content. When the herbal medicine is added to the chemical oscillation system, the interference to the induction phase and the oscillation phase may be different from the effects of various substances in the oscillation system, causing different changes. By comparing the different changes of the E-t curve after adding different herbal medicines to the chemical oscillation system, not only the corresponding E-t curve is completely different from the curve of the blank system under the same experimental conditions, but more importantly, it reflects the chemical composition characteristics of different herbal medicine (Jin and Shen, 2017; Mohtashami et al., 2018). The fingerprint can be used to analyze the chemical composition of herbal medicine as a whole and to identify or qualitatively analyze herbal medicine. Electrochemistry has been widely used for sensing purpose in pharmaceutic fields (Fu et al., 2019; Fouladgar et al., 2020; Karimi-Maleh et al., 2020b; Mohanraj et al., 2020; Xu et al., 2020; Ying et al., 2020; Zabihpour et al., 2020). In this work, we proposed an electrochemical oscillation system based on B-Z reaction. The effect of a combination of each reagent, reaction temperature, and stirring speed on the induction period, oscillating period, and oscillating life were optimized. The nuts of *Prunus persica, Prunus davidiana*, and *Prunus armeniaca* have been selected as samples.

## Materials and Methods

All chemicals were analytical grade and used without purification. The nuts of *P. persica, P. davidiana*, and *P. armeniaca* were purchased from a local pharmacy and grounded into a powder. The chemical oscillating reaction is carried out in a continuously stirred jacketed reactor. The experimental temperature is controlled at 310 K. A certain amount of herbal machines was added into the discussed reactor with different concentrations of H_2_SO_4_ (10 ml), CH_3_COCH_3_ (5 ml), and MnSO_4_ (5 ml). The mentioned solution was stirred 10 min before injection of 5-ml KBrO_3_. The E-t curve was monitored all the time until potential oscillation disappears. All electrochemical experiments were recorded using a CHI760E working station.

## Results and Discussion

**Figure 1:**
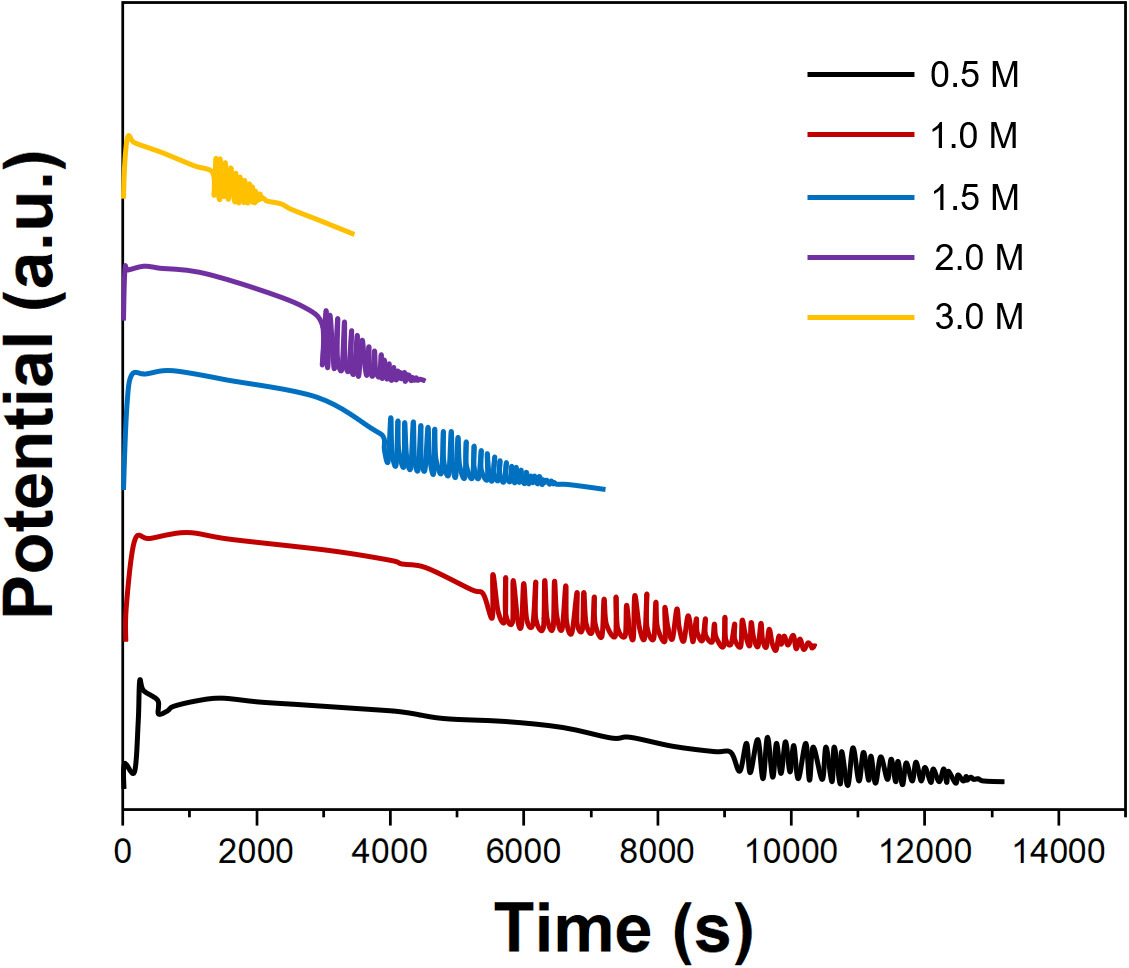
Oscillating profiles of *Prunus persica* recorded using different concentrations of H_2_SO_4_.

*Prunus persica* has been used for optimizing the concentrations of all reagents used in the B-Z reaction. Figure 1 shows the effect of the H_2_SO_4_ concentration on the electrochemical oscillation of *P. persica*. It can be seen that the oscillation profiles recorded after injection of 10 ml of H_2_SO_4_ with different concentrations were different. When the sulfuric acid concentration was 1.0 M, the oscillation life is the longest, and the amplitude was the largest. When the sulfuric acid concentration exceeded 2.0 M, the oscillations decayed faster. When the sulfuric acid concentration was 0.5 M, the induction period of the oscillating reaction was too long. Therefore, we selected 1.0 M of sulfuric acid for an oscillating reaction.

**Figure 2:**
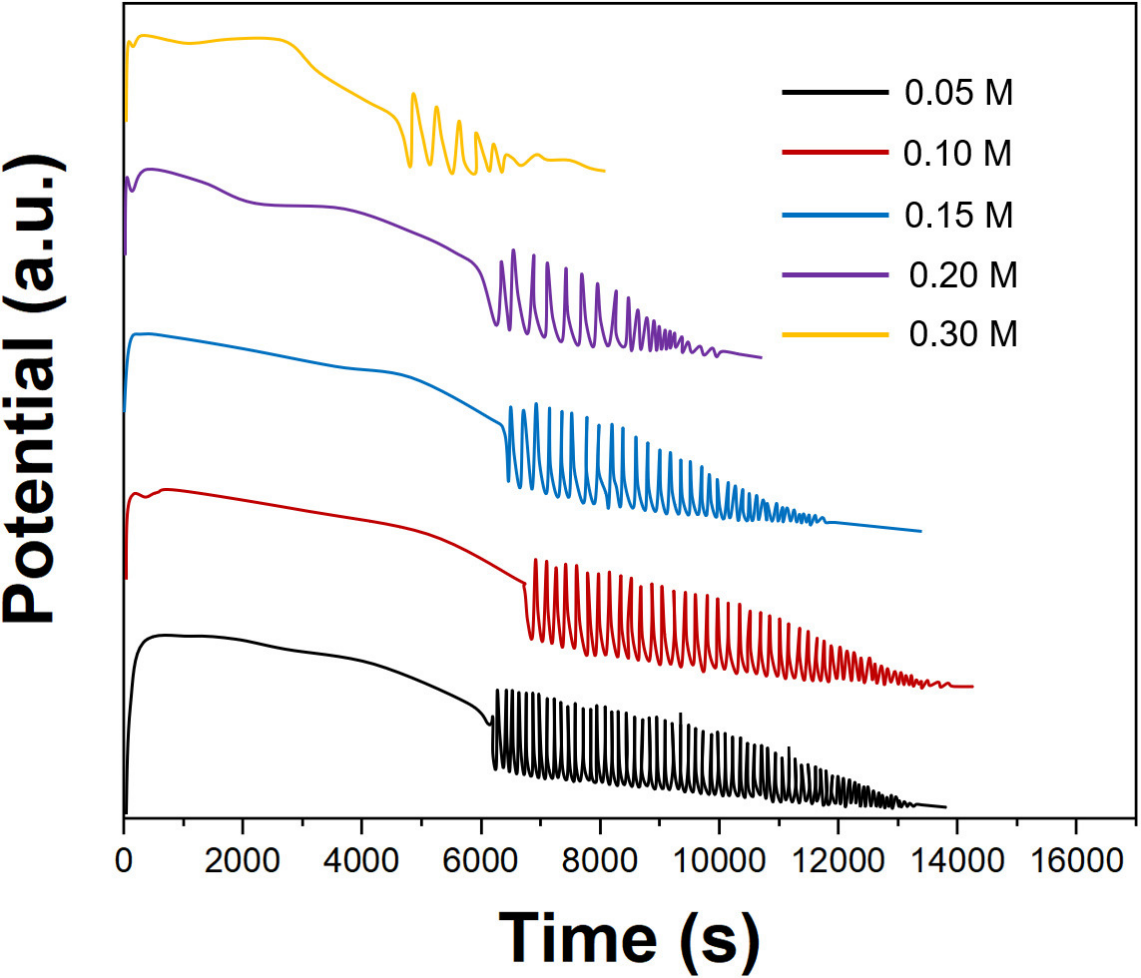
Oscillating profiles of *Prunus persica* recorded using different concentrations of MnSO_4_.

Figure 2 shows the effect of the MnSO_4_ concentration on the electrochemical oscillation of *P. persica*. In this case, manganese ions were catalysts for oscillating reactions and had little effect on amplitude and induction time. Therefore, we selected 0.1 M of MnSO_4_ for an oscillating reaction.

**Figure 3:**
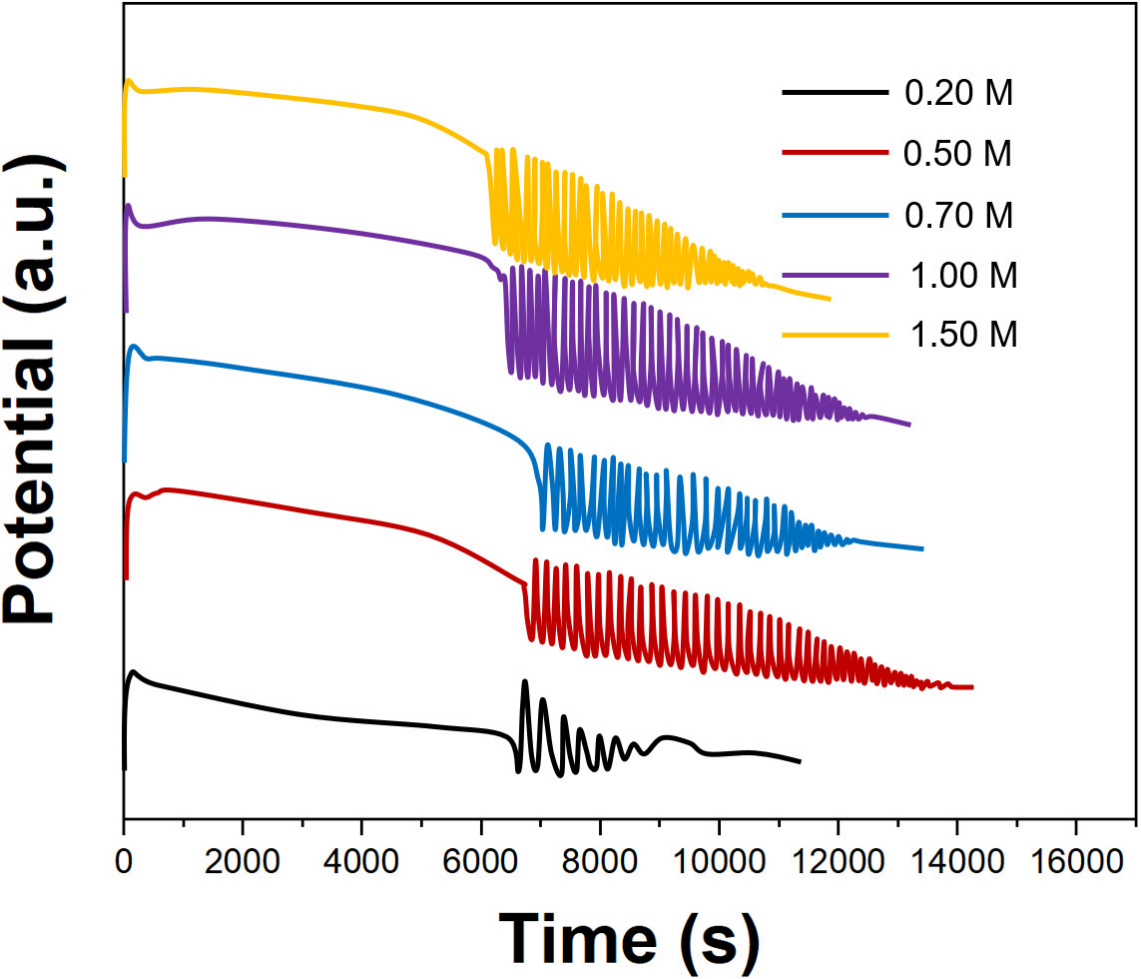
Oscillating profiles of *Prunus persica* recorded using different concentrations of CH_3_COCH_3_.

Figure 3 shows the effect of the CH_3_COCH_3_ concentration on the electrochemical oscillation of *P. persica*. When the concentration of CH_3_COCH_3_ was 0.2 M, the oscillation reaction is irregular; when the concentration of CH_3_COCH_3_ reaches 0.5 M, a regular oscillation reaction can be produced. CH_3_COCH_3_ can also produce normal oscillation response at higher concentrations. The role of CH_3_COCH_3_ was to react with Br_2_ to form CH_3_COCH_2_Br to remove excess Br_2_. CH_3_COCH_2_Br is a toxic preparation, which is irritating to the eyes, so we selected 0.5 M of CH_3_COCH_3_ for an oscillating reaction.

**Figure 4:**
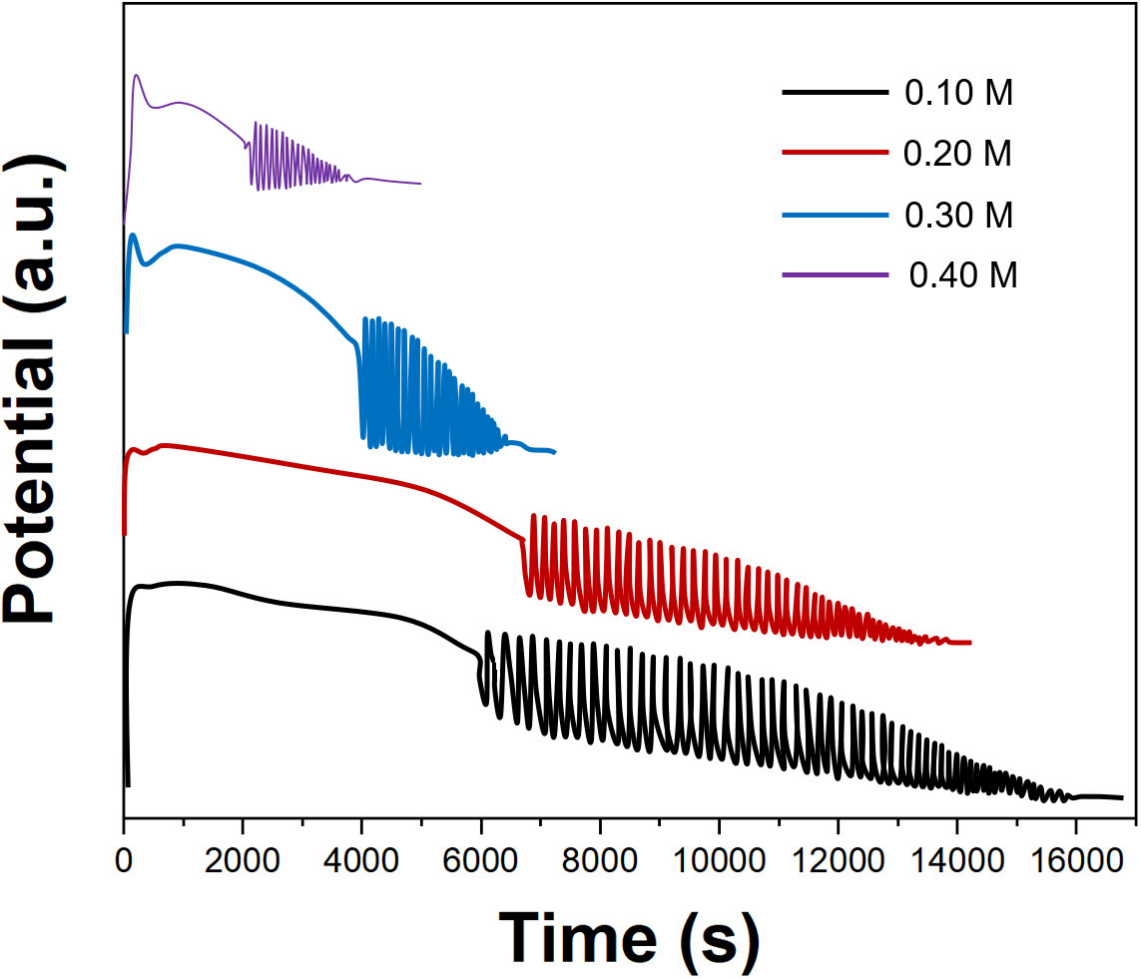
Oscillating profiles of *Prunus persica* recorded using different concentrations of KBrO_3_.

Figure 4 shows the effect of the KBrO_3_ concentration on the electrochemical oscillation of *P. persica*. It can be seen from Figure 4 that as the KBrO_3_ concentration increases, the maximum amplitude, induction time, and oscillation life increase. However, the solubility of KBrO_3_ at room temperature is small, so we selected 0.3 M of KBrO_3_ for an oscillating reaction.

**Figure 5:**
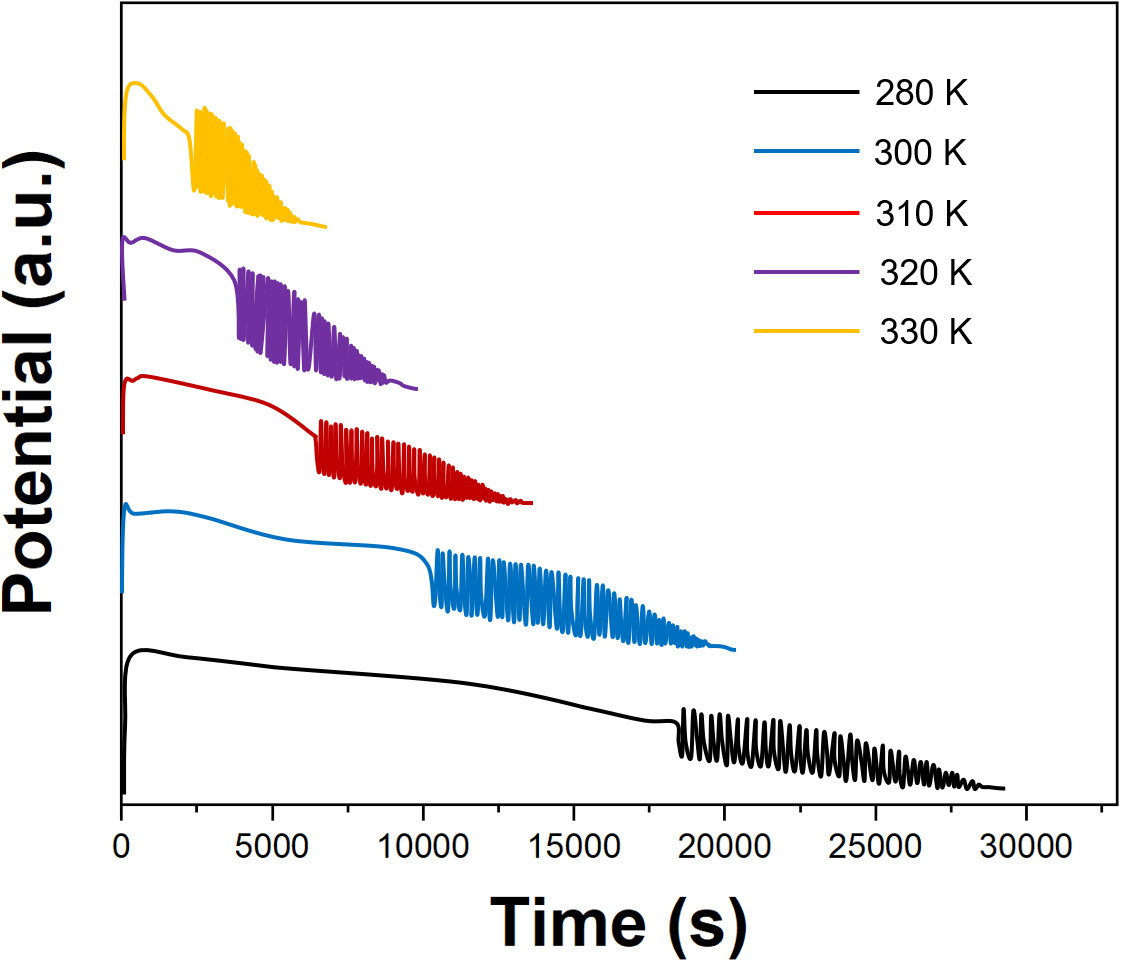
Oscillating profiles of *Prunus persica* recorded using different temperature.

Figure 5 shows the effect of the temperature on the electrochemical oscillation of *P. persica*. The results show that as the temperature increases, the fingerprint data change in different patterns. Generally speaking, the higher the temperature, the longer the induction time, the shorter the oscillation period, the shorter the oscillation lifetime, and the larger the amplitude. Therefore, the selection of appropriate temperature conditions is of great significance for the control of reaction time, the stability of the fingerprint, and the reproducibility. Therefore, we selected 310 K for oscillating reactions.

**Figure 6:**
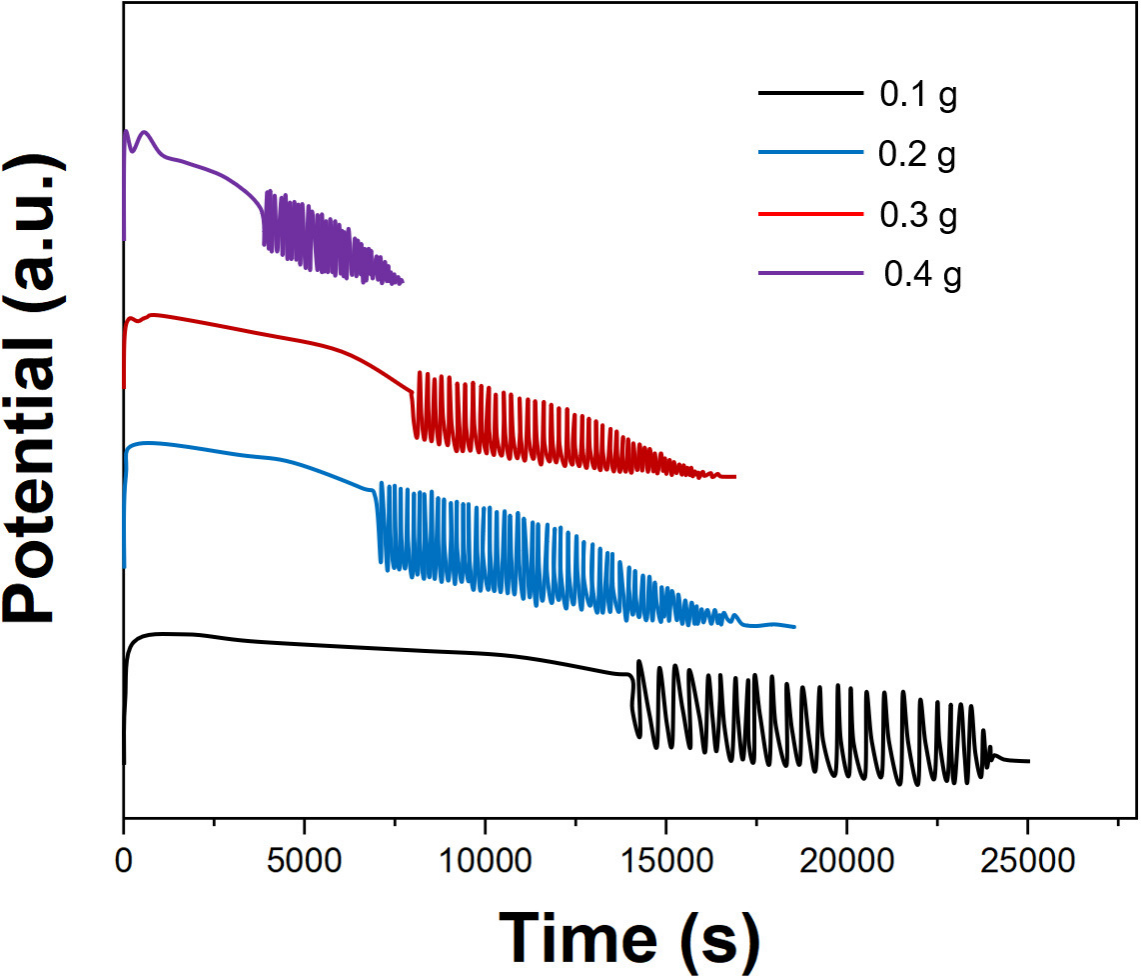
Oscillating profiles recorded using different amount of *Prunus persica*.

Figure 6 shows the effect of the amount of *P. persica* on the electrochemical oscillation. When *P. persica* was 0.1 g, the substrate that can be used as the B-Z oscillation reaction was the least, so the induction period was the longest, and the oscillation period was also the longest. When its oscillating substrate was consumed, its oscillation suddenly stops. With the increase in the dosage of herbal medicine, the number of chemical components in the herbal medicine participating in the oscillation reaction also increased. The oscillation reaction was more likely to occur, so the reaction rate was accelerated, the induction period was reduced, and the oscillation period and oscillation life were also reduced. When the dosage of herbal medicine exceeds 0.5 g, the front part of the curve becomes irregular, which was not conducive to the acquisition of characteristic information. When the dosage was small, the reaction time will become too long, requiring a longer analysis time. To obtain better information, the amount of herbal medicinal materials used in the fingerprints recording was 0.3 g.

The reproducibility of electrochemical oscillation patterns for *P. persica* has been studied. The results indicated that under the same experimental conditions, the various indexes of the electrochemical oscillation measured are basically the same, RSD ≤ 2%, indicating good reproducibility.

**Figure 7:**
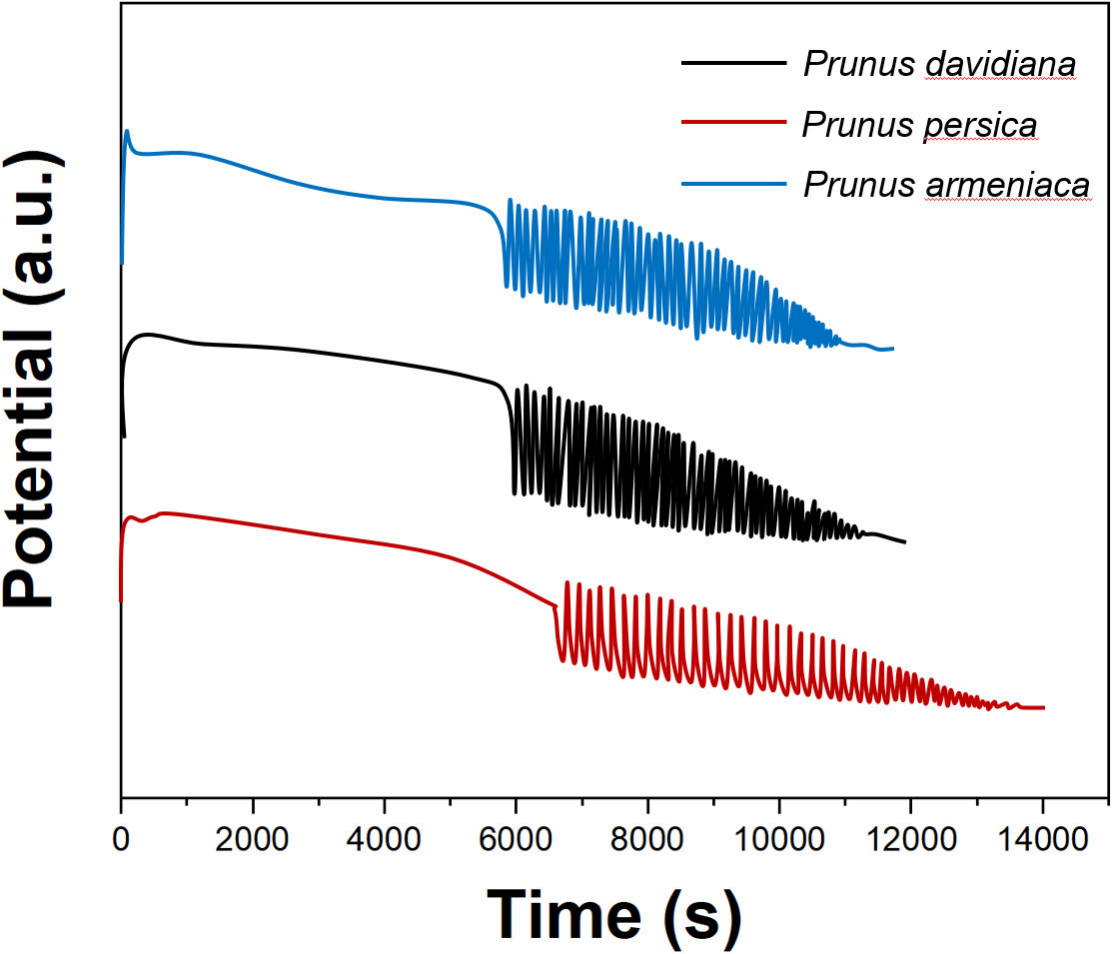
Oscillating profiles recorded for *Prunus persica, Prunus davidiana*, and *Prunus armeniaca*.

Figure 7 is the electrochemical fingerprint of *P. persica, P. davidiana*, and *P. armeniaca*. It can be seen that due to the different chemical components contained in different species, their participation in the oscillating reaction process is different, so the E-t curve induction time, oscillation life, maximum potential, start-up potential, and other data are different. At the same time, the shape of the induction curve and the oscillation curve are also different. These features help identify species.

**Figure 8:**
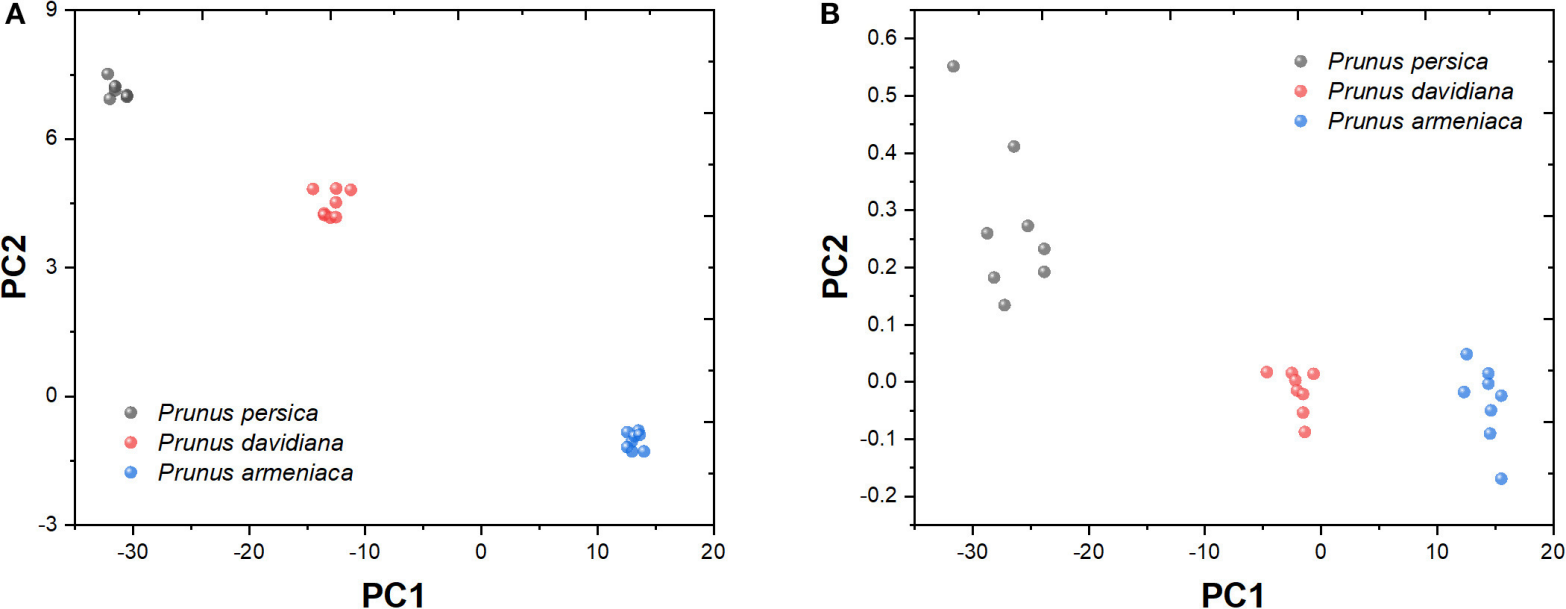
Principal component analysis of *Prunus persica, Prunus davidiana*, and *Prunus armeniaca*. **(A)** PC1 vs. PC2. **(B)** PC1 vs. PC3.

Figure 8 shows the principal component analysis of *P. persica, P. davidiana*, and *P. armeniaca* recorded from different samples. Table 1 shows the characteristic value and contribution value of data after the principal component analysis. The contribution rates of the first three principal components were 89.44, 7.25, and 3.31%, respectively. From the analysis in Figure 8A, it can be seen that the three samples can be distinguished obviously, whereas part of the data in Figure 8B is mutually charged.

**Table 1:** Eigenvalues and contributions of principle component.

**PCA**	**Eigenvalue**	**Contribution rate (%)**	**Accumulating contribution rate (%)**
PC1	0.588	89.44	89.44
PC2	0.624	7.25	96.69
PC3	0.007	3.31	100

**Figure 9:**
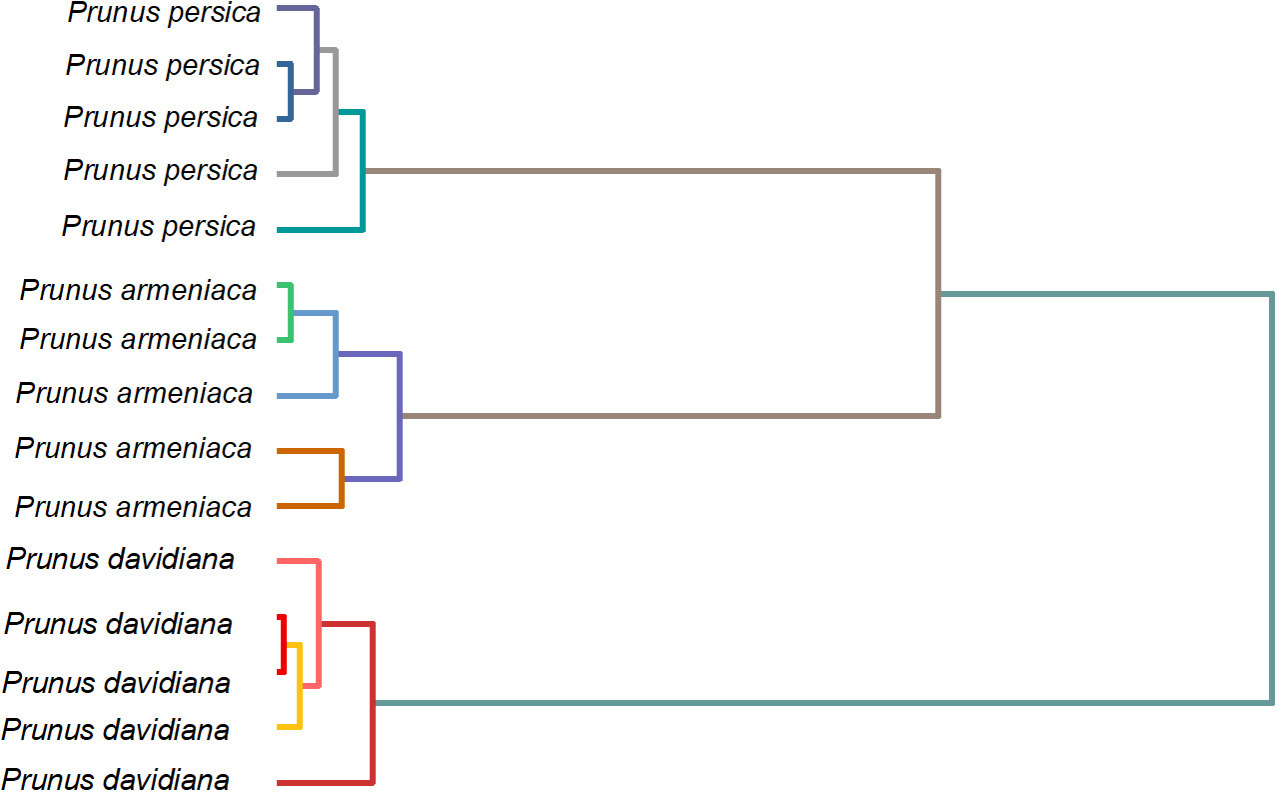
Do9oendrogram of *Prunus persica, Prunus davidiana*, and *Prunus armeniaca* deduced from the fingerprint recorded using B-Z reaction.

As the oscillating fingerprint of species is positively correlated with the distribution and amount of chemical compounds, we attempted to use the fingerprint, as mentioned earlier, for dendrogram analysis. Figure 9 shows the dendrogram of *P. persica, P. davidiana*, and *P. armeniaca* deduced from the fingerprint recorded using B-Z reaction. The dendrogram was divided into three main principal infrageneric clades. Each cluster only contains one species, suggesting that no outlier was observed in this study. Based on the discussed results, we proposed a simple method for herbal medicines identification.

## Conclusion

In this work, we proposed an authentication method based on the electrochemical oscillation system. B-Z reaction has been selected due to its high stability. The electrochemical oscillation profiles of *P. persica, P. davidiana*, and *P. armeniaca* were recorded. The recorded profile varies between the species due to the presence of different contents of chemical compounds. Based on the recorded oscillation fingerprint, these species can be effectively identified. Also, the dendrogram results suggest no outlier was observed in this study. Therefore, the proposed electrochemical oscillation can be used for herbal medicines identification.

## Data Availability Statement

The original contributions presented in the study are included in the article/supplementary material, further inquiries can be directed to the corresponding author/s.

## Author Contributions

SY, YY, and XW contributed conception and design of the study. LS and MH conducted electrochemical experiments. SY, XW, and TZ performed the statistical analysis. XW and SY wrote the manuscript. All authors contributed to manuscript revision, read, and approved the submitted version.

## Conflict of Interest

The authors declare that the research was conducted in the absence of any commercial or financial relationships that could be construed as a potential conflict of interest.
